# Cardiac Syndrome X: The Sensitive Heart of a Young Adult Man

**DOI:** 10.7759/cureus.20669

**Published:** 2021-12-24

**Authors:** Shikha Jha

**Affiliations:** 1 Internal Medicine, Saint Peter’s University Hospital, New Brunswick, USA

**Keywords:** anginal chest pain, coronary artery angiography, healthy young man, microvascular angina, cardiac syndrome

## Abstract

Cardiac syndrome X (CSX), now well known as microvascular angina, is a mysterious cardiac condition in medical science. While the symptoms suggest obstructive coronary disease, the actual angiography turns out to be negative or nonsignificantly obstructive. Despite being a benign condition, its presence increases the risk of adverse cardiovascular events and leads to poor quality of life in the patients. The prevalence of cardiac syndrome X is higher in women, mostly in postmenopausal states. This case report sets a different clinical picture of cardiac syndrome X, where a young male patient is found to have this syndrome. A 38-year-old male went to the hospital with a chief complaint of substernal chest pain for one hour. An electrocardiogram (EKG) showed nonspecific ST-T wave changes, and the cardiac troponin results were nonsignificant. On the contrary, the myocardial perfusion scan came back positive for significant ischemia in various parts of the heart. The patient underwent a coronary angiogram, which showed normal coronary arteries. In view of similar chest pain episodes in the past and the presence of risk factors, he was discharged with extensive counseling on lifestyle modification and medical management. This case report raises awareness about this syndrome's classic clinical scenario and chronology of events in a rare class of the population. Through this case report, clinicians can learn the art of diagnosing this syndrome and provide appropriate patient care in near-miss situations.

## Introduction

Cardiovascular diseases are one of the most common health concerns in the world. Out of all the cardiovascular conditions, coronary artery disease (CAD) is among the most serious ones. Yet, up to 20%-30% of patients presenting with chest discomfort characteristic of angina demonstrate no signs of obstructive CAD, defined as ≥50% stenosis in at least one major coronary artery, upon angiography [1]. Cardiac syndrome X has been largely replaced by microvascular angina, and it is diagnosed when the pathogenesis is unknown (without epicardial artery stenosis or abnormal flow reserve). Cardiac syndrome X (CSX) is more common in women than in men [2]. A 2015 study of about 1500 such individuals suggests that the prevalence may be as high as 67% [3]. The management of this condition should primarily comprise understanding its mechanism. Thus, diagnostic tests should aim at identifying the cause of the symptoms in the individual patient, i.e., myocardial ischemia, increased pain perception, abnormalities of adrenergic tone, noncardiac mechanisms, etc. [4]. Patients with cardiac syndrome X have an overall good prognosis. Hence, the main goal should be toward educating the patient about this syndrome and enhancing their quality of life.

## Case presentation

**Figure 1:**
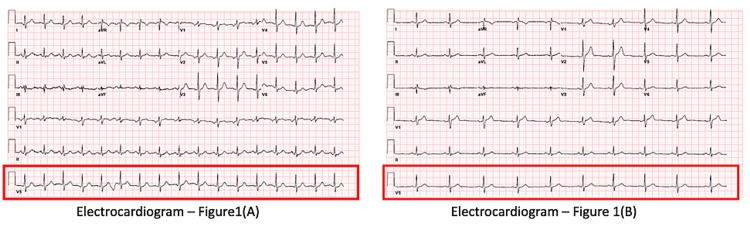
Electrocardiogram: (A) before the nitroglycerine treatment and (B) after the nitroglycerine treatment

The patient is a 38-year-old male who presented to the emergency room with a chief complaint of chest pain. The chest pain was in the substernal region, squeezing and pressure type in character, non-radiating, and worse with exertion that lasted for about an hour. He reports having recurrent chest pain in the last few years. His family history was pertinent for heart diseases in several family members. His social history was significant for his smoking history of 15 pack-years. On presentation, his vitals were pertinent for having tachycardia of 100 beats per minute. Physical examination was pertinent for having a high body mass index (37.2 kg/m^2^), first and second heart sounds present, no murmurs and gallops, and peripheral pulses palpable. The electrocardiogram (EKG) is pertinent for nonspecific ST-T wave changes and sinus tachycardia with a ventricular rate of 106 beats per minute (Figure 1(A)). He was given two doses of sublingual nitroglycerine, which remarkably relieved his chest pain. After the nitroglycerine treatment, a repeat electrocardiogram was done, which showed resolution of the nonspecific ST-T wave changes (Figure 1(B)).

He received a loading dose of aspirin and statin in the emergency department. The laboratory investigations were significant for abnormal lipid profiles (Table 1).

**Table 1:** Laboratory investigations

Laboratory investigation	Result
Cardiac troponin I	<0.03 ng/mL (three times six hours apart)
D-Dimer	<150 ng/mL
TSH	1.690 uIU/mL
HbA1c	4.8%
Total cholesterol	215 mg/dL (high)
Triglyceride	110 mg/dL
Direct HDL cholesterol	33 mg/dL (low)
Direct LDL cholesterol	154 mg/dL (high)

**Figure 2:**
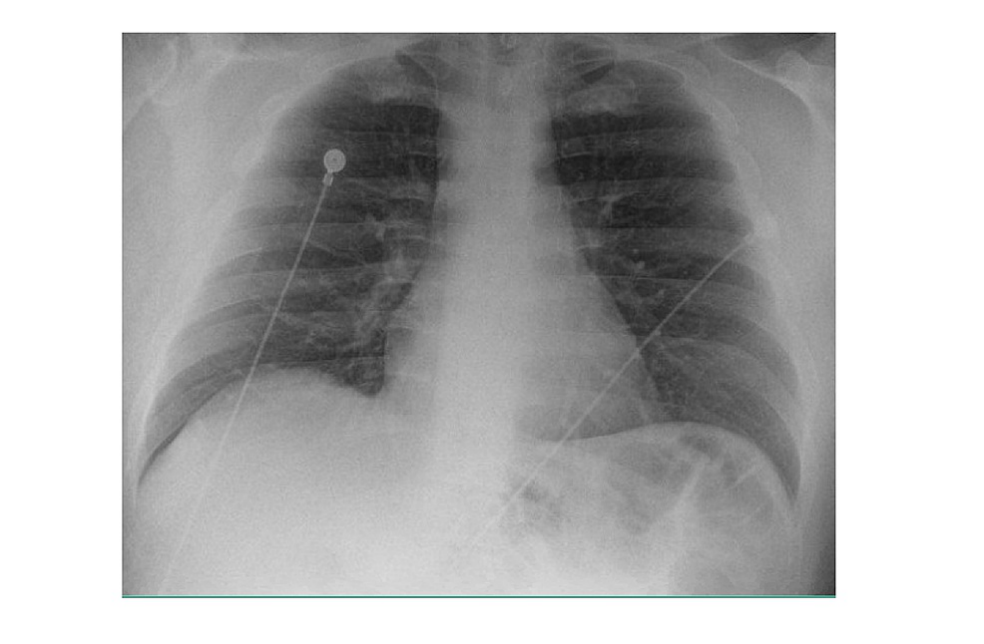
Chest X-ray (anterior-posterior view): no evidence of cardiopulmonary abnormality

The chest X-ray did not show any evidence of focal consolidation, pulmonary edema, or pneumothorax (Figure 2).

**Figure 3:**
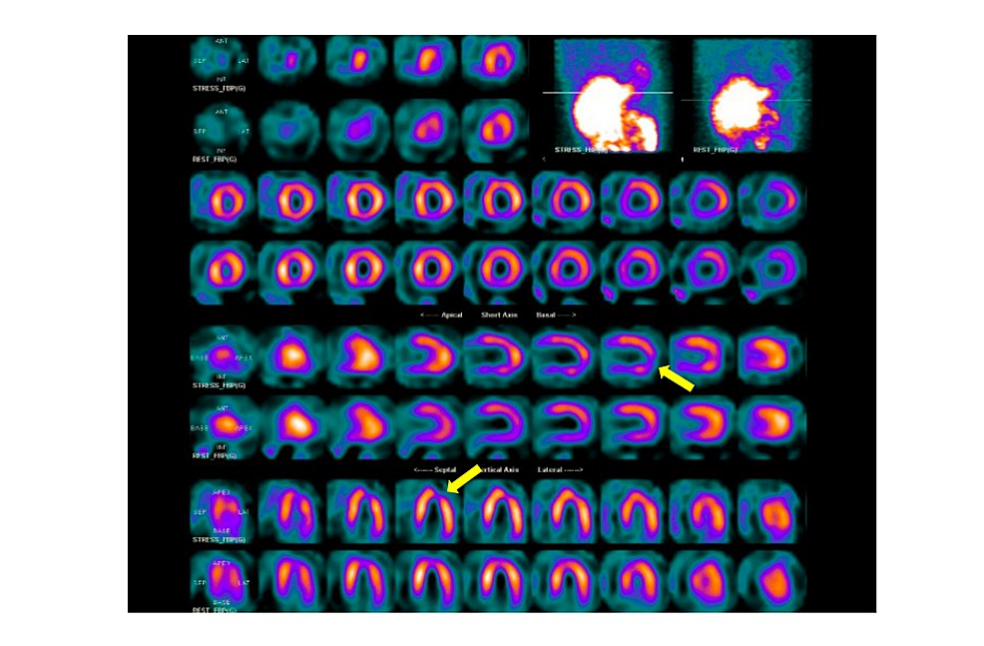
Myocardial perfusion scan: yellow arrows indicate ischemic areas

The cardiology team was consulted by the emergency department. The team recommended that the patient should undergo a myocardial perfusion scan, in view of multiple risk factors and EKG changes. The myocardial perfusion scan (Lexiscan) showed a left ventricular ejection fraction of 60%. There were findings suggesting mild ischemia of the distal lateral wall and apical lateral segment regions (Figure 3).

**Figure 4:**
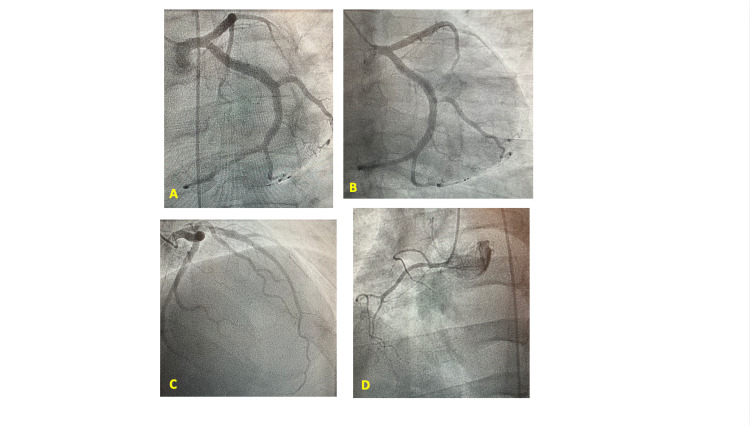
Coronary angiogram: (A) left main coronary artery, (B) left circumflex, (C) left anterior descending, and (D) right coronary artery

In view of the abnormal Lexiscan findings, the cardiology team recommended further evaluation with cardiac catheterization. The patient underwent cardiac catheterization, which showed near-normal coronary arteries (Figure 4).

The patient was explained about the cardiac catheterization results and the phenomena of his medical condition. He was counseled about the importance of weight loss, healthy eating habits, and the benefits of quitting smoking. He was discharged on sublingual nitrates and angiotensin-converting enzyme inhibitors for the treatment of further episodes of similar chest pain. He was also given atorvastatin for lipid management. He was asked to follow up with his primary medical doctor and the cardiology team closely post-discharge.

## Discussion

Cardiac syndrome X comprises typical chest pain and normal coronary arteries on angiogram. It is a heterogeneous syndrome, which is mostly attributable to a noncardiac cause [4]. A fraction of patients may have angina secondary to the transient diminishing of myocardial blood flow [4]. One of the most common diagnostic tests that point toward an ischemic etiology is a defect in the perfusion seen on the myocardial scan. Dysfunction of the endothelium is the most prominent cause of microvascular aberration in these patients. Some of the most common risk factors for this aberration are smoking, diabetes mellitus, hyperlipidemia, and hypertension. A thorough history taking, physical examination, and resting echocardiogram is a must to assess for obstructive coronary artery disease. The electrocardiogram (EKG) is usually normal between episodes [5]. Transient changes include ST-segment depression with anginal pain, but the absence of EKG changes does not exclude a cardiac etiology because of the low sensitivity of the EKG [6].

Most stable patients with chest pain with characteristics of myocardial ischemia will be referred for stress testing with or without radionuclide perfusion imaging or echocardiography [7]. In some patients with chest pain, coronary angiography is indicated for further diagnostic testing or as a prelude to revascularization. The coronary angiogram will show normal epicardial coronary arteries or mild coronary artery disease (<30% stenosis) [8]. Patients with cardiac syndrome X have an excellent long-term clinical prognosis. Unfortunately, a notable percentage of patients experience either symptom progression or further recurrent episodes [9]. There is the deterioration of quality of life due to the persistent episodes of precordial pain, hospital readmissions, and repeated diagnostic tests resulting in a significant consumption of resources [10].

Cardiac syndrome X (CSX) is treated with nonpharmacological and pharmacological methods. The nonpharmacological method mainly focuses on lifestyle modification [10], such as healthy eating habits, intake of a nutritious diet, adequate exercise, appropriate weight loss, cessation of smoking, and alcohol consumption. The pharmacological step includes anti-anginal medications such as calcium channel blockers, beta-blockers, and nitrates. Other commonly used medications include angiotensin-converting enzyme inhibitors. Aspirin and statin can be given to patients with evidence of atherosclerotic cardiovascular disease not responsible for angina or those at high risk [11]. Exercise capacity can be improved by a physical training program [12]. Behavioral therapies have proven to be effective in the reduction of chest pain, over three to six months intervals [13]. Other beneficial techniques include enhanced external counterpulsation (EECP) [14]. If resistant to all other treatments, spinal cord stimulation could be an option for quality of life improvement in these patients [15].

## Conclusions

Cardiac syndrome X is, by history, a mystifying syndrome that requires a clear understanding of the diagnostic pattern. This medical condition prevails commonly in day-to-day clinical practice. As clinicians, we need to have a thorough understanding of this syndrome, as it can be easily missed, especially if it exists in the less likely affected population of young male patients. Thorough communication with the patients about the nature of this disease and its potential cardiovascular long-term risk is crucial. The management is a stepwise approach, based on the patient’s response to both nonpharmacological and pharmacological treatment, followed by close outpatient follow-up.
